# The Philadelphia Polyclinic

**Published:** 1896-09

**Authors:** 


### THE PHILADELPHIA POLYCLINIC.

To the Editor:—We send herewith a copy of our announcement. You will see that our Faculty has been increased and our facilities enlarged, especially in the matter of laboratory equipment. We have an excellent Rœntgen apparatus, which is daily used for diagnosis and study.

Our new department of Neuro-Pathology is unexcelled, and some valuable researches have been published. Our dispensary service has increased until now it is the largest of any teaching institution in Philadelphia, running up to nearly 16,000 new patients annually. Many of these represent rare and obscure diseases, being sent by their physicians from a distance.

The number of major operations performed at the Polyclinic Hospital during the past year was over five hundred, of which a great number were abdominal sections. Our pupils have the opportunity of seeing probably twice as many more in the private hospital practice of the staff.

We trust that the announcement will be found worthy of notice in your pages.

Very respectfully,
THE PHILADELPHIA POLYCLINIC.

The editor has received the anouncement above referred to, and most cordially recommends it as a finely-prepared book, containing a well-prepared statement of the advantages of this college. For those who may not be familiar with it, we will state that *The Philadelphia Polyclinic* is a college where are admitted as students only those who have graduated from recognized schools of medicine. Its teaching is of the old or "regular" school of medicine, but its students are graduates of the homœopathic as well as the dominant school. It is admirably appointed for special instruction in diagnosis and surgery, as a perusal of the beautiful photographic views of its interior to be found in the announcement will abundantly testify. Physicians desiring to improve their knowledge of diagnosis should enter this school. Send for a copy of their announcement. Address Max J. Stern, M. D., Secretary Philadelphia Polyclinic, Lombard Street, West of Eighteenth, Philadelphia, Pa. —ED.

